# Renal Effects of Hyperbaric Oxygen Therapy in Patients with Diabetes Mellitus: A Retrospective Study

**DOI:** 10.1155/2021/9992352

**Published:** 2021-06-12

**Authors:** Martin Sedlacek, Nicole P. Harlan, Jay C. Buckey

**Affiliations:** ^1^Section of Nephrology, Dartmouth-Hitchcock Medical Center, Lebanon, NH 03756, USA; ^2^Section of Hyperbaric Medicine, Dartmouth-Hitchcock Medical Center, Lebanon, NH 03756, USA; ^3^Geisel School of Medicine, Section Chief, Section of Hyperbaric Medicine, Dartmouth-Hitchcock Medical Center, Lebanon, NH 03756, USA

## Abstract

Hyperbaric oxygen therapy (HBOT) is an adjunctive treatment for patients with diabetic foot ulcers. The prolonged high oxygen level used in HBOT can produce oxidative stress, which may be harmful to the kidney. Animal experiments suggest HBOT does not harm renal function and may have an antiproteinuric effect, but little is known on the effect of HBOT in humans. We performed a retrospective chart review of 94 patients with diabetes mellitus who underwent HBOT at our institution over an eight-year period. Thirty-two patients had serum creatinine levels within 60 days of the start and the end of treatment. Creatinine levels were 1.41 ± 0.89 mg/dl before and 1.52 ± 1.17 mg/dl after hyperbaric treatments with no statistically significant difference (mean (postcreatinine + precreatinine/2) = 0.10 mg/dl, SE = 0.11, *t* = 0.89). Twenty-three patients had proteinuria measurements before and after HBOT mainly by urine dipstick analysis. A Wilcoxon signed-rank test showed less proteinuria after HBOT than before (*N* = 23, *p*=0.002). Proteinuria was absent in 7 of 23 patients (30%) before HBOT and 13 of 23 patients (57%) after HBOT, a reduction by almost 50%. This observation is remarkable because oxidative stress might be expected to increase rather than decrease proteinuria.

## 1. Introduction

Clinical practice guidelines recommend using hyperbaric oxygen therapy (HBOT) adjunctively for patients with Wagner grade 3 or higher nonhealing diabetic foot ulcers [1]. Patients may receive up to 40 consecutive daily treatments with 100% oxygen given at pressures above atmospheric pressure (usually 2 to 2.4 times the sea level pressure) [2]. HBOT is believed to exert some of its beneficial effects on wound healing by increasing reactive oxygen radical production and angiogenesis. These same factors are implicated in the pathogenesis of microvascular complications of diabetes in the eye and in the kidney [3]. This suggests HBOT might be potentially harmful for patients with diabetes; however, animal studies show reduced proteinuria with HBOT treatments [4–6]. Virtually, no clinical data exist on the effect of HBOT on proteinuria and renal function in patients with diabetes mellitus, despite its widespread use in these patients. We performed a retrospective chart review of patients with diabetes undergoing HBOT for various indications at our institution to assess the changes in both creatinine levels and proteinuria with HBOT. We hypothesized that HBOT might increase proteinuria due to the repeated, high-level oxygen exposures.

## 2. Methods

The Dartmouth–Hitchcock Institutional Review Board (IRB) approved this retrospective chart review, and collected data were deidentified as mandated. A list of all patients with diabetes mellitus at the Dartmouth–Hitchcock Medical Center who underwent hyperbaric oxygen therapy (HBOT) was obtained from the institutional HBOT registry [7]. Data on proteinuria, serum creatinine, use of angiotensin-converting enzyme inhibitors (ACEIs), and use of angiotensin receptor blockers (ARBs) was obtained from electronic medical records. Patients who had fewer than 10 HBOT were excluded, as we reasoned that at least 10 treatments would be required to see any effects. Patients on dialysis were excluded as their parameters reflect mainly dialysis timing. Patients who had measurements of serum creatinine within 60 days prior to starting and after ending HBOT and/or had an assessment of proteinuria before and after HBOT were included. We considered a 60-day period for creatinine both practical and reasonable as progression of diabetic nephropathy is measured in years rather than months [8]. The development of macroalbuminuria (>30 mg/dl) usually signals irreversible progression to diabetic nephropathy [8]. Proteinuria is an important marker of progression of chronic kidney disease (CKD) used for staging [9], and consequently, we included all available before and after proteinuria measurements in our study. As per the 2012 KDIGO guidelines on CKD staging, urine dipstick (Chemstrip 10 COBAS, Roche) and spot urine protein to creatinine ratios were used for proteinuria assessment when microalbuminuria measurements were not available [9]. Quantitative proteinuria data were converted into dipstick proteinuria which was defined according to the specifications of Chemstrip 10 COBAS, Roche as trace (>6 mg/dl), 1+ (30 mg/dl), 2+ (100 mg/dl), or 3+ (500 mg/dl). A negative test strip result or a negative microalbuminuria or protein/creatinine ratio on a spot urine test was defined as no proteinuria. Urine dipstick specific gravity data were classified as either concentrated (SG >1.010) or isosthenuria/dilute (SG ≤1.010).

JMP 7 (SAS Institute) and MATLAB® 2020a (The MathWorks, Inc) were used for data analysis. Serum creatinine data were analyzed using paired Student's *t*-test, and the Wilcoxon signed-rank test was used to detect differences in proteinuria and specific gravity before and after HBOT.

## 3. Results

Over eight years from September 2011 to November 2019, 94 patients with diabetes mellitus were referred for HBOT with promoting wound healing as the most frequent indication. Thirty-eight patients were excluded from analysis because they had fewer than 10 HBOT treatments. Twenty-four additional patients were excluded because of insufficient data or because they underwent dialysis treatments. Thirty-two patients were included in the study. They had between 10 and 60 treatments, breathing 100% oxygen at either 2.0 or 2.4 atmosphere absolute (ATA) for 60–90 minutes once a day (Table 1). There was no control group in this retrospective study.

**Figure 1:**
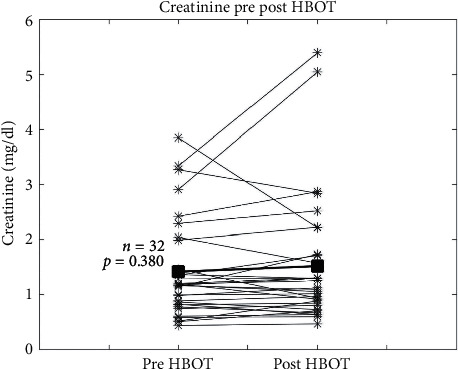
Creatinine levels before and after hyperbaric oxygen therapy (HBOT). Measurements were taken within 60 days of starting and ending HBOT. Asterisks show the individual results, and the squares are the mean. The paired Student's t-test *p* value was used to obtain the *p* value.

There was no difference (mean (postcreatinine + precreatinine/2) = 0.10 mg/dl, SE = 0.11, *t* = 0.89 *p*=0.38) between serum creatinine levels before HBOT (1.41 ± 0.89 mg/dl) and after HBOT (1.51 ± 1.67 mg/dl) per matched pair analysis (Figure 1). In the 12 patients with abnormal renal function before HBOT (eGFR <60 ml/min), there also was no statistically significant difference (mean (postcreatinine + precreatinine/2) = 0.18 mg/dl, SE = 0.28, *t* = 0.66, *p*=0.52) in creatinine between before (2.21 ± 0.90 mg/dl) and after HBOT (2.40 ± 1.40 mg/dl).

**Figure 2:**
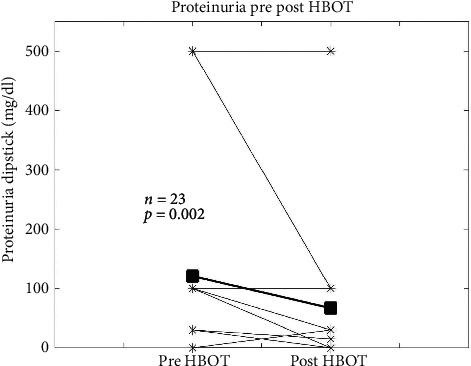
All available proteinuria levels before and after hyperbaric oxygen therapy (HBOT). Asterisks show the individual results, and the squares are the mean. The Wilcoxon signed-rank test was used to calculate the *p* value.

Urine protein measurements before and after HBOT were available in 19 patients in the form of a urine test strip result and in 6 patients as microalbuminuria or spot urine protein/creatinine ratio. Because of overlap, there were 23 patients in total, who had measurements of proteinuria. The median time from urine test to HBOT in days±SE was 14 ± 17 days before HBOT and 18 ± 27 days after HBOT. A Wilcoxon signed-rank test showed that there was less proteinuria after HBOT than before (*N* = 23, *p*=0.002) (Figure 2). Indeed, proteinuria was absent in 7 of 23 patients (30%) before HBOT and 13 of 23 patients (57%) after HBOT, a reduction by almost 50%. To assess whether negative dipstick proteinuria findings may have been due to dilution, we used the Wilcoxon signed-rank test to analyze dipstick specific gravity data. There was no difference in urine specific gravity in the urine before and after HBOT (*n* = 12, *p*=0.248).

The proportion of patients treated with angiotensin-converting enzyme inhibitors (ACEIs) or angiotensin receptor blocker (ARB) was 50% in patients with and without proteinuria before and after HBOT.

## 4. Discussion

Although HBOT exposes patients to high levels of oxygen daily, which likely produces oxidative stress, this observational study showed no evidence for kidney damage.

Most data on the effect of HBOT on kidney function come from animal experimentation. Verma et al. found a dose-dependent decrease in urinary albumin excretion and albumin/creatinine ratio in an HBOT-treated db/db diabetic mouse model [4]. Yilmaz et al. found that HBOT has a synergistic effect with an angiotensin receptor blocker to reduce proteinuria in the adriamycin rodent model of focal segmental glomerulosclerosis (FSGS) [5]. Sonmez et al. found the same with an angiotensin-converting enzyme inhibitor [6]. Several studies in rat models of ischemia perfusion injury found beneficial effects of HBOT on histological and biochemical markers of injury. Ramalho et al. found attenuated negative effects of ischemic/reperfusion injury on creatinine levels and proteinuria in rats treated with HBOT [10]. Interestingly, there was a tendency toward decreased proteinuria in their sham-operated group that underwent HBOT, consistent with a potential antiproteinuric effect of HBOT. Not all studies show a benefit, however. A study of HBOT in a rat model of gentamycin-induced acute kidney injury (AKI) found no benefit [11]. A study of HBOT in a rat model of rhabdomyolysis-induced AKI also found no benefit [12]. Berkovitch et al. found no difference in renal pathology, serum creatinine, and cystatin C between rats that underwent HBOT and a control group [13] and concluded HBOT was not harmful to the kidney. On the other hand, a study of the effects of HBOT in normal New Zealand White rabbits found increased serum creatinine levels and histologic signs of proximal tubular damage in the treatment group [14].

The only study of the effects of HBOT on renal function in humans was a preliminary report published by Harrison et al. of a study of HBOT on urine biomarkers in 17 volunteer patients with diabetes; however, this report did not include any clinical data on renal function and proteinuria [3].

In our retrospective study of eight years of HBOT in diabetic patients, we found no evidence of adverse renal effects. Patients exposed to established mechanisms of renal injury are more at risk if their renal function is abnormal at baseline. Similarly, one might expect a more pronounced potential adverse effect of HBOT on renal function in patients who had decreased renal function prior to exposure to HBOT. We did not find an adverse effect on renal function in the 12 patients with abnormal renal function at baseline. Moreover, we found an apparent decrease in proteinuria, consistent with animal data [4–6]. Urine dipstick tests are widely used to screen for proteinuria because they are of low cost, simple, and rapid. They were the most frequent form of proteinuria assessment in our data. A recent NIDDK- and NKF-sponsored meta-analysis of large international trials found that the urine dipstick category of trace and more proteinuria has a relatively low sensitivity of 62% but a high specificity of 87.8% for detecting clinically significant albumin-to-creatinine ratios of 30 mg/g or greater. In the higher dipstick protein category ++, the sensitivity and specificity were greater at 77.6% and 97.5% [15]. This is because dipstick testing does not adjust for urine concentration and is less sensitive detecting proteinuria in dilute urine than measurement of albumin-to-creatinine ratio. We did not find significant differences in urine concentration before and after HBOT, which argues that the observation of lesser proteinuria is not an artifact of dilution. The most common hemodynamic side effect of HBOT is a transient increase in blood pressure (BP), likely due to effects on nitric oxide during HBOT [16]. As the normal variation of physical activity, posture, temperature, and consequently, blood pressure is responsible for diurnal variations of proteinuria [17], one might have expected an increase in proteinuria because of increased BP with HBOT treatments.

Oxidative stress with increased production of reactive oxygen species and dysregulation of VEGF are pathogenic mechanisms in early diabetic nephropathy [18, 19]. High levels of oxygen with HBOT might be expected to increase proteinuria, but we found the opposite. In an apparent paradox, HBOT can reduce oxidative stress over time and influence VEGF by mechanisms that are incompletely understood [3]. To date, there has not been evidence of worsening of ischemia-reperfusion injury when HBO is used in settings such as compromised grafts and flaps. In those settings, oxygen is reintroduced into ischemic tissue. Similarly, enhanced oxygen delivery might be beneficial in ischemic zones in the diabetic kidney. Another hypothetical explanation for the observation of a reduction of proteinuria with HBOT could be through a generalized reduction of inflammation associated with successful foot ulcer or wound treatment.

General antioxidant approaches in diabetic nephropathy have failed in large trials [18, 20]. This makes the finding of a possible proteinuria reducing effect of HBOT remarkable, as it may constitute a first description of an intervention on the redox systems that reduces proteinuria in patients. Our observation calls for confirmation in a prospective study and further investigation of its mechanism.

## Figures and Tables

**Table 1:** Characteristics of the patients.

*Numerical data*			
	Mean	SD	Range
Age	65 years	+/− 11 years	40–90 years
Number of HBOT	28 treatments	+/− 14 treatments	10–60 treatments
Days of HBOT	47 days	+/− 30 days	4–157 days

*Categorical data*			
Gender	9 female	23 male	
On ACEI/ARB	Yes in 16 patients	No in 16 patients	
Type of diabetes	31 patients type 2DM	1 patient type 1 DM	
ATA	25 patients 2.4 ATA	7 patients 2.0 ATA	
Treatment time	90 min in 31 patients	60 min in 1patient	

*Indications for HBOT*	19 patients (60%)	Wound healing	
	4 patients (13%)	Radiation necrosis	
	3 patients (9%)	Radiation cystitis	
	3 patients (9%)	Compromised flap	
	1 patient (3%)	Crohn's disease	
	1 patient (3%)	Vasculitis	
	1 patient (3%)	Severe anemia	

## Data Availability

Deidentified data are available on request from the Section of Hyperbaric Medicine at Dartmouth–Hitchcock Medical Center.
